# Early versus Delayed Feeding after Percutaneous Endoscopic Gastrostomy Placement in Children: A Meta-Analysis

**DOI:** 10.3390/children7090124

**Published:** 2020-09-03

**Authors:** Jun Watanabe, Kazuhiko Kotani

**Affiliations:** Division of Community and Family Medicine, Jichi Medical University, Shimotsuke-City, 3311-1 Yakushiji, Shimotsuke-City, Tochigi 329-0498, Japan; m06105jw@jichi.ac.jp

**Keywords:** child, diet, enteral nutrition, gastrostomy, pediatric practice

## Abstract

Early feeding after percutaneous endoscopic gastrostomy (PEG) placement is an accepted practice in the treatment of adult patients and the knowledge is clinically extrapolated in the treatment of children. To verify this treatment in children—as there are some specific features of PEG-related practices in children—the present study aimed to review meta-analyses of early feeding (within 4 h) after PEG placement in children. We searched the PubMed database for articles published until July 2020. A quality assessment was performed using the Grading of Recommendations, Assessment, Development, and Evaluation method. Three randomized controlled trials (208 patients) were eligible for inclusion. No patients died within 72 h. Early feeding resulted in little to no difference in the length of hospital stay (mean difference [MD] −7.47, 95% confidence interval [CI] −25.16 to 10.21; I^2^ = 95%) and vomiting events (risk ratio 0.84, 95% CI 0.55 to 1.31; I^2^ = 0%). In a subgroup analysis, early feeding without antibiotics reduced the length of hospital stay in one study (MD −21.60, 95% CI −22.86 to −20.34) but early feeding with antibiotics did not affect the length in two studies (MD 0.28, 95% CI −6.49 to 7.06; I^2^ = 0%). Overall, the certainty of the evidence was not very high. In summary, early feeding after PEG placement may be a safe alternative to delayed feeding in children. The findings in children seemed similar to those in adults, while there is a need for further studies that specifically investigate PEG placement-related practices in children.

## 1. Introduction

Childhood is a dynamic period of growth and development [1,2]. Eating disorders, dysphagia, and failure to thrive can lead to malnutrition [1,2]. In consideration of health maintenance and/or disease management, percutaneous endoscopic gastrostomy (PEG) is a method to provide nutrition into the stomach through the abdominal wall for patients who have a functioning gastrointestinal tract but who are unable to eat sufficiently [3]. Originally, PEG was described in the treatment of pediatric patients and its use in postoperative feeding spread worldwide [4]. Approximately 4% of all patients who require PEG in the United States are children [5]. More understanding of PEG for children is necessary among patients, caregivers, and medical staff [6,7]. They should know some differences in the indications of PEG between adults and children, and in the associated complications and prognosis; for instance, PEG placement is often indicated for cerebrovascular disease and malignancy. In adults [8], while it is commonly indicated for neurological and neuromuscular disorders in children [9,10,11]. The mortality rate at 30 days after PEG placement was reported to be 5.5–23.9%, which is low in children relative to in adults [12,13,14].

Recent meta-analyses of studies involving adults revealed that early feeding after PEG placement can be a safe alternative to delayed feeding [15,16]. Early feeding is generally considered to be defined as feeding within 4 h, based on national data in the United States (92% of patients begin feeding from 4–24 h after PEG placement) [17]. The guidelines of the American Society for Parenteral and Enteral Nutrition then recommended nutritional support by early feeding within 4 h after PEG placement in both adults and children [18]. The findings regarding early feeding after PEG placement have mainly been drawn from the studies of adult populations [15,16]. Although the guidelines included two random controlled trials (RCTs) in children [19,20], it appeared that children were not analyzed with meta-analytic methods separately from adults. Due to the differences in the indications, complications, and prognosis between adults and children who undergo PEG placement [8,9,11,12,13,14], the analyses of PEG placement can be restrictedly considered in children.

Accordingly, the present study aimed to investigate the clinical suitability of early feeding versus delayed feeding after PEG placement in a population restricted to children based on meta-analyses. The findings of children may reinforce or arrange the current status that this knowledge regarding early feeding after PEG placement in adults is extrapolated to children.

## 2. Materials and Methods

### 2.1. Statement on Review

The present study accorded with the Preferred Reporting Items for Systematic Reviews and Meta-Analyses (PRISMA) Statement [21].

### 2.2. Literature Search Strategy

The PubMed database was searched for relevant studies published from 1946 to July 2020. The following search terms were used: (“Gastrostomy” [Mesh] OR “Enteral Nutrition” [Mesh] OR “Intubation, Gastrointestinal” [Mesh] OR “Gastrostomy”[tiab] OR “Enteral nutrition”[tiab] OR “intubation gastrointestinal”[tiab]) AND (“early”[tiab] OR delay*[tiab] OR “feeding”[tiab]). All RCTs comparing the outcomes of early (within 4 h) versus delayed feeding after PEG placement in children were included. The outcomes were mortality within 72 h, the length of hospital stay (hours), vomiting events, and adverse events, while referring to previous meta-analyses of adult populations [15,16]. Articles that did not analyze a child population or which were not written in the English language were excluded. The reference lists of guidelines were searched [18,22]. The references were hand-searched and were included if appropriate.

### 2.3. Data Collection and Assessment of Risk of Bias

Two reviewers (J.W. and K.K.) independently performed all data collection and risk assessments, and the disagreement between the two reviewers was resolved by discussion. The titles and abstracts for inclusion were screened. The full text of potentially eligible studies was subsequently screened. The reviewers abstracted the published data and compared results. The following study characteristics were extracted, according to previous meta-analyses of adult populations [15,16]: Trial authors, study location, antibiotics, participants number, mean age, time/timing of feeding, and follow-up period. The reviewers assessed the risk of bias in each eligible study, using version 2 of the Cochrane risk-of-bias tool for randomized trials (RoB2) tool [23]. Each potential source of bias was graded as high, low, or unclear.

### 2.4. Data Management

In the meta-analysis, dichotomous data were analyzed as the risk ratio (RR) and 95% confidence interval (CI); continuous data were analyzed as the mean difference (MD) and 95% CI. The mean and standard deviation (SD) of continuous data were analyzed based on the method of the Cochrane Handbook [23]. SD was substituted from other studies when the studies did not report SD [24]. Adverse events associated with PEG placement were summarized based on the definitions of the original article.

### 2.5. Assessment of Heterogeneity

Heterogeneity was assessed by a visual inspection of forest plots and using the I^2^ statistic (I^2^ values of 0–40%: Might not be important; 30–60%: May represent moderate heterogeneity; 50–90%: May represent substantial heterogeneity; 75–100%: May represent considerable heterogeneity) [23]. Reasons for heterogeneity were assessed using a subgroup analysis of patients with and without antibiotics when there was substantial heterogeneity (I^2^ >50%). When <10 studies were identified, the publication bias was not assessed by a visual inspection of the funnel plot and Egger test according to the Cochrane handbook [23].

### 2.6. Data Synthesis

A random-effects meta-analysis was performed using RevMan version 5.4 (The Cochrane Collaboration, Copenhagen, Denmark). A summary of findings table was created with the primary and secondary outcomes. The quality of evidence was determined using the Grading of Recommendations, Assessment, Development, and Evaluation (GRADE) method [25].

## 3. Results

### 3.1. Study Selection

**Figure 1:**
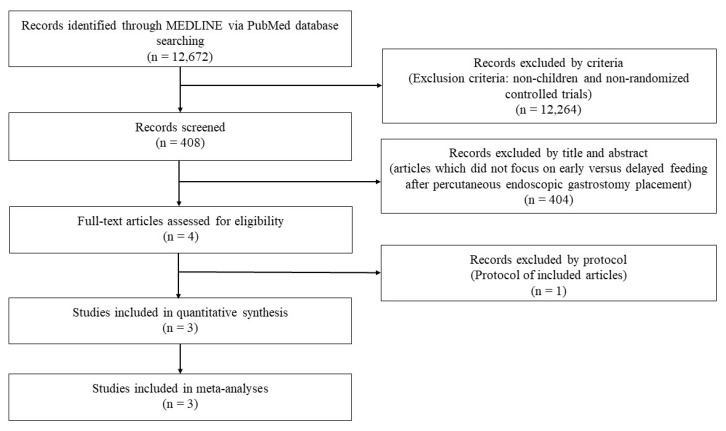
Flow chart of article selection.

Figure 1 shows the flow of the selection of articles that compared early versus delayed feeding after the PEG placement in children. A total of 12,672 records were identified, and a total of three RCTs met the inclusion criteria [19,20,26]. Thus, three studies with 208 patients were included in the meta-analysis [19,20,26].

### 3.2. Study Characteristics

**Figure 2:**
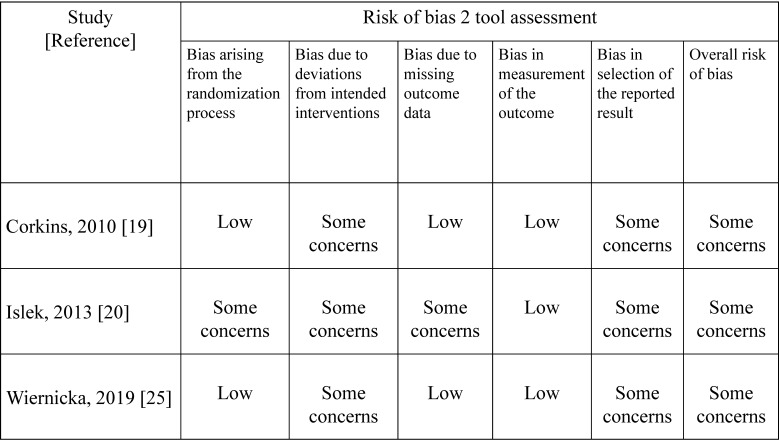
Risk of bias graph and table for mortality within 72 h.

**Figure A1:**
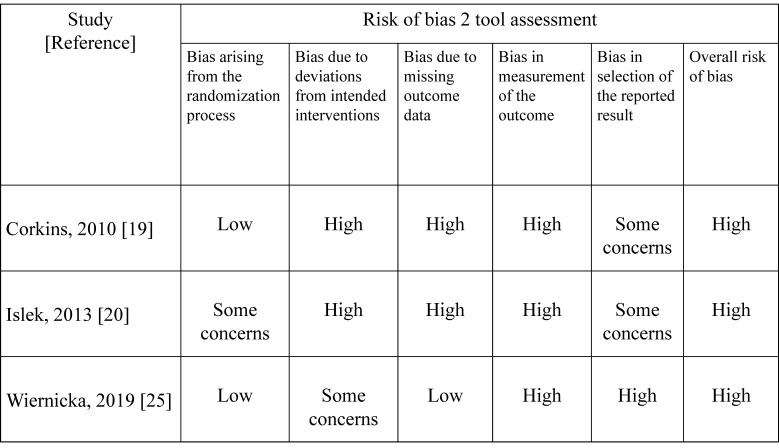
Risk of bias for hospital stay.

**Figure A2:**
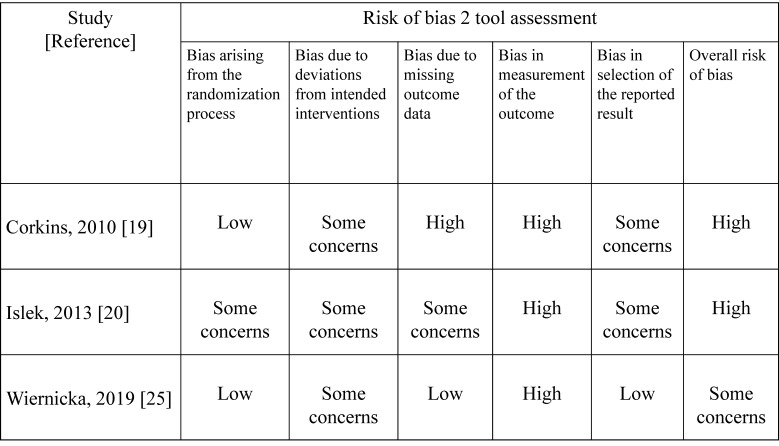
Risk of bias for vomiting events.

**Figure A3:**
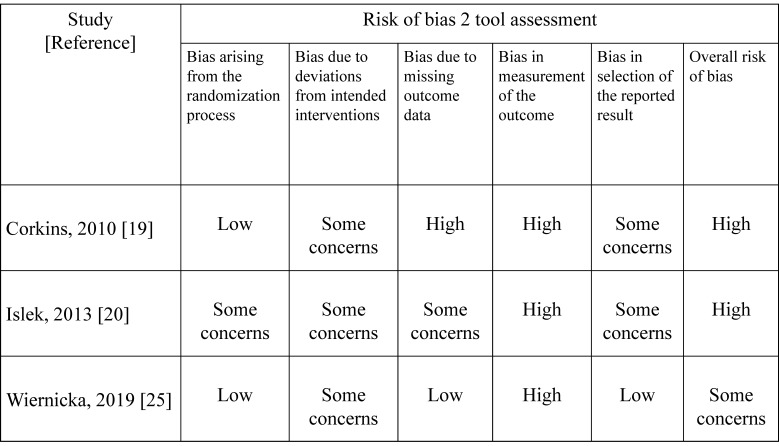
Risk of bias for adverse events.

Table 1 shows a summary of the characteristics of the reviewed studies [19,20,26]. Early feeding was defined as within 3 h in two studies and within 4 h in one study. Preoperative antibiotic administration was performed in two studies, but not performed in one. The median age in the included studies was 5.17 (range; 1.48 to 7.25) years. The risk of bias in each study is shown in Figure 2 and Appendix A Figure A1, Figure A2 and Figure A3. Overall, there were some concerns regarding the risk of bias for mortality.

### 3.3. Outcomes

#### 3.3.1. Mortality

None of the patients in the three studies died within 72 h after PEG placement [19,20,26].

#### 3.3.2. Length of Hospital Stay

**Figure 3:**
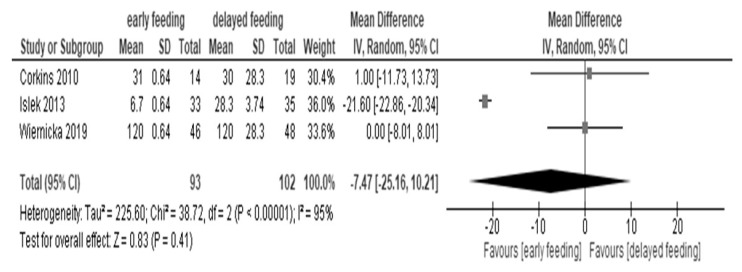
Forest plot of hospital stay (hours).

Similar median hospital stays (five days) were seen in both early and delayed feeding [19,20,26]. The effect of early feeding on the length of hospital stay (hours) after PEG placement showed little to no difference in comparison to delayed feeding (195 patients): MD −7.47 h, 95% CI −25.16 to 10.21; I^2^ = 95%) (Figure 3) [19,20,26].

**Figure A4:**
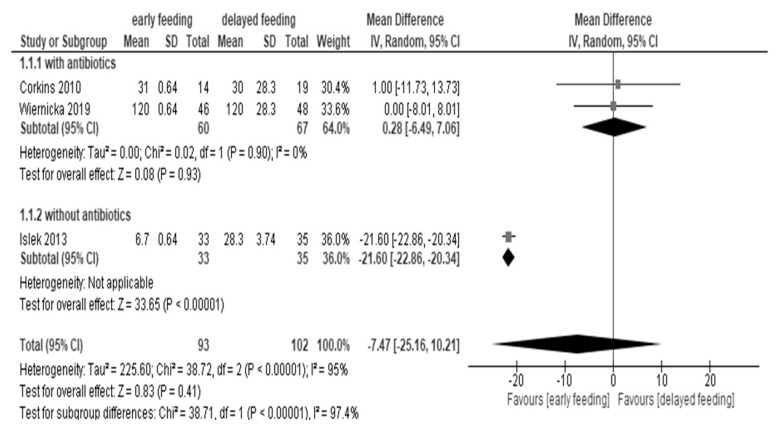
Forest plot of the hospital stay for children managed with and without antibiotics.

In a sub-analysis, one study showed that early feeding without antibiotics resulted in a slight reduction in the length of hospital stay relative to delayed feeding (MD −21.60 h, 95% CI −22.86 to −20.34) [20], while early feeding with antibiotics did not affect the length of stay in two studies (MD 0.28 h, 95% CI −6.49 to 7.06; I^2^ = 0%) (Appendix A Figure A4) [19,26].

#### 3.3.3. Vomiting Events

**Figure 4:**
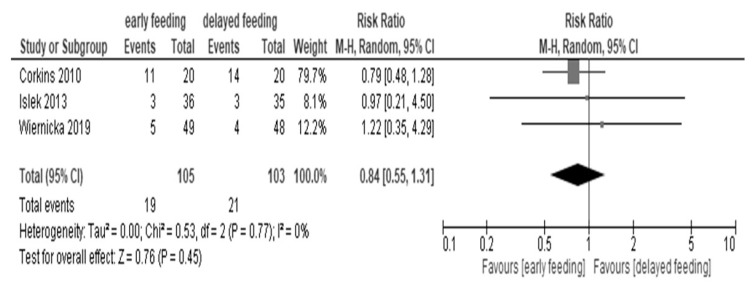
Forest plot of vomiting events.

The effect of early feeding after PEG placement on vomiting events showed little to no difference in comparison to delayed feeding (206 patients): RR 0.84, 95% CI 0.55 to 1.31; I^2^ = 0%; very low certainty of evidence) (Figure 4) [19,20,26].

#### 3.3.4. Adverse Events

Three studies reported adverse events [19,20,26]. In the study by Corkins et al., one patient in the delayed feeding group developed a fever that did not require antibiotic treatment [19]. In the study by Islek et al., leakage from gastrostomy fistula, peritonitis, and aspiration were not observed; the only adverse event reported was vomiting (as described above) [20]. In the study by Wiernicka et al., complications occurred in 13 patients (25.5%) in the early feeding group and 18 patients (37.5%) in the delayed feeding group [26]. In the delayed feeding group, two patients underwent laparotomy and one patient died seven days after PEG placement (this death was not related to the procedure). The other complications, such as fever and redness around the gastrostomy, were mild and did not require treatment.

#### 3.3.5. Certainty of Evidence

The certainty of evidence was moderate for mortality within 72 h, as a result of imprecision due to the small sample size. The certainty of evidence for hospital stay was very low as a result of imprecision due to the small sample size and a high risk of bias due to the selection of the reported results, and inconsistency that was considered to represent considerable heterogeneity. The certainty of evidence was low for vomiting events and adverse events due to imprecision and high risk of bias (Table 2).

## 4. Discussion

The results of the present review of three RCTs [19,20,26] revealed that early feeding within 4 h after PEG placement in child patients had little to no effect on mortality, hospital stay, or vomiting events in comparison to delayed feeding. The findings were similar to the general consensus that was mainly reached from the studies of adult populations [15,16]. Namely, the verification of findings in a population restricted to children suggested that the findings regarding early feeding after PEG placement in adults could be clinically extrapolated to children, even though there were differences between children and adults with regard to the indications for PEG, complications, and prognosis [8,9,11,12,13,14].

In recent meta-analyses of adult studies, the mortality within 72 h did not differ between early feeding and delayed feeding groups [15,16]. Twelve deaths (3.9%)—four (2.6%) in early feeding and eight (5.2%) in delayed feeding—were reported in another meta-analysis in adults [15]. In the present review of studies of children, there were no deaths within 72 h after PEG placement. The differences in mortality between adults and children may be due to the fact that children undergoing PEG placement had better prognostic factors than the adults (children can be presumed to have fewer underlying diseases and highly preserved organ functions) [12,13,14]. Early feeding after PEG placement in children may be thus a safe alternative to delayed feeding, as it is in adults.

Early feeding did not markedly affect the length of hospital stay in recent meta-analyses of studies in adults as well as the present review in children [15,16]. The hospitalizations unrelated to PEG placement (which could need a long stay) were beforehand excluded in the protocol of RCTs included in the present review (in fact, the total number analyzed was fewer as in Figure 3 than in Figure 4) [19,20,26] and this might be a reason for the no effect of early feeding on the length of hospital stay. The use of antibiotics for PEG placement may be partly related to the reason. A recent systematic review described that the administration of prophylactic antibiotics in patients undergoing PEG placement reduced peristomal infection, and the guidelines also recommend the use of prophylactic antibiotics [21,27]. The review analyzed only the patients over 16 years of age [21]. In the sub-analysis of our present review, early feeding showed little or no effect on the hospital stay in children who received antibiotics, while it might have shortened the hospital stay in one study that analyzed children who underwent PEG placement without antibiotics [20]. The expectation of early feeding to shorten the hospital stay may be offset in patients who receive antibiotics because the preoperative administration of antibiotics reduces the incidence of infection but alters the favorable immune function of gut microbiota [28,29]. Further studies are needed to determine whether this is a child-specific phenomenon.

Meta-analyses of adult studies revealed that vomiting events did not differ between early feeding and delayed feeding groups [15,16]. In the present review of studies involving children, similarly, there was little to no difference in vomiting events between the early feeding and delayed feeding groups. The mechanism of gastroesophageal reflux disease, which causes vomiting after PEG placement is partly associated with changes in the angle of His or increased gastric distension with perturbation of the lower esophageal sphincter, and this is seen in children [30,31]; however, vomiting events can improve on their own or be treated with medical therapy in most children [32,33]. This may explain the less significant effects of feeding on these events in children.

Meta-analyses of studies involving adults revealed that early feeding did not increase the rate of complications requiring treatment, such as gastrostomy fistula leakage, peritonitis, and aspiration, in comparison to delayed feeding [15,16]. In the present review, two studies reported that there were almost no complications and one study reported that the incidence of complications was 25% in the early feeding group and 37% in the delayed feeding group. Up to one-third of children who underwent PEG placement had mild complications; the incidence of severe complications was much lower [2]. Previous studies of adult populations reported that gastric residual volumes were not associated with complications [34]. The two studies in child populations reported that gastric residual volumes were low and there was no difference between the early and delayed feeding groups [19,20]. Thus, the gastric residual volume appears to be unrelated to complications in children. Additionally, the preoperative diagnosis, indications, prematurity, and neuropathy did not affect the rate of postoperative complications [35]. Thus, early feeding does not seem to be associated with complications requiring treatment, although physicians must be prudent to detect the development of complications.

The findings from the present review should be interpreted with caution because the certainty of the evidence was low or very low, with the exception of the certainty of evidence related to mortality, which was moderate. The main reason for the downgrading of the evidence levels was imprecision due to the small sample size. As outcomes, length of hospital stay, vomiting events, and adverse events tended to be associated with a high risk of bias, resulting in a low or very low certainty of evidence. Even so, early feeding was generally considered as safe as delayed feeding because the certainty of evidence in relation to mortality was moderate. 

The present meta-analysis was associated with several limitations. Although the three studies included in the review were all RCTs, the number of eligible studies was small and the studied populations were also relatively small. The high risk of bias for outcomes other than mortality meant that there were deviations from the intervention and outcome data. In future studies, the outcomes, including adverse events, should be clearly defined and the entire population should be followed, with blinded assessors. Further dedicated RCTs are needed to draw definitive conclusions.

## 5. Conclusions

The present meta-analysis demonstrated that introducing early feeding within 4 h after PEG placement in children may be as safe as delayed feeding, as it is in adults. Future studies are warranted to establish better quality evidence to support early feeding after PEG placement in children.

## Figures and Tables

**Table 1:** Summary of the characteristics of the included studies.

Authors [Reference]	Year	Country	Antibiotics	Feeding	Time (Hours)	Patient Number	Age (Years)
Corkins [19]	2010	U.S.A.	Yes	Early	3	20	1.48
				Delayed	6	20	1.49
Islek [20]	2013	Turkey	No	Early	4	36	5.29
				Delayed	12	35	5.17
Wiernicka [25]	2019	Poland	Yes	Early	3	49	5.58
				Delayed	8	48	7.25

**Table 2:** Summary of findings.

Early versus Delayed Feeding after Percutaneous Endoscopic Gastrostomy Placement in Children						
Patient or Population: Children Setting: After Percutaneous Endoscopic Gastrostomy Placement Intervention: Early Feeding Comparison: Delayed Feeding						
Outcomes	Anticipated Absolute Effects * (95% CI)		Relative Effect (95% CI)	Patient Number (Studies)	Certainty of the Evidence (GRADE)	Comments
	Risk with Delayed Feeding	Risk with Early Feeding				
Mortality follow-up: Three days	not pooled	not pooled	not pooled	208 (3 RCTs)	Moderate ^a^	Three studies reported no deaths.
Hospital stay	-	MD 7.47 h lower (25.16 to 10.21)	-	195 (3 RCTs)	Very low ^a,b^^,^^c^	The evidence about the effect of early feeding on hospital stay is very uncertain.
Vomiting events	181 per 1000	152 per 1000 (100 to 237)	RR 0.84 (0.55 to 1.31)	208 (3 RCTs)	Low ^a,b^	The evidence suggests that early feeding does not increase vomiting events.
Adverse events	There were 26.0% adverse events in the early feeding and 34.3% adverse events in the delayed feeding.		-	208 (3 RCTs)	Low ^a,b^	Three studies reported adverse events and all adverse events were mild.

CI: Confidence interval; MD: Mean difference; RR: Risk ratio; RCT: randomized control trial. * The risk in the intervention group (and its 95% CI) is based on the assumed risk in the comparison group and the relative effect of the intervention (and its 95% CI). GRADE Working Group grades of evidence: High certainty: We are very confident that the true effect lies close to that of the estimated effect. Moderate certainty: We are moderately confident in the estimated effect. The true effect is likely to be close to the estimated effect, but there is a possibility that it is substantially different. Low certainty: Our confidence in the estimated effect is limited: The true effect may be substantially different from the estimated effect. Very low certainty: We have very little confidence in the estimated effect. The true effect is likely to be substantially different from the estimated effect. ^a^ Downgraded because of imprecision due to the small sample size. ^b^ Downgraded because of high risk of bias due to the selection of the reported results. ^c^ Downgraded because of inconsistency as considered to represent considerable heterogeneity.
